# Inhibition of Corneal Neovascularization by Topical and Subconjunctival Tigecycline

**DOI:** 10.1155/2014/452685

**Published:** 2014-08-17

**Authors:** Sertan Goktas, Ender Erdogan, Rabia Sakarya, Yasar Sakarya, Mustafa Yılmaz, Muammer Ozcimen, Nejat Unlukal, Ismail Alpfidan, Fatih Tas, Erkan Erdogan, Abdulkadir Bukus, Ismail Senol Ivacık

**Affiliations:** ^1^Department of Ophthalmology, Konya Training and Research Hospital, Meram, 42090 Konya, Turkey; ^2^Department of Histology and Embryology, Selcuk University, 42030 Konya, Turkey; ^3^Department of Histology and Embryology, Mevlana University, 42030 Konya, Turkey

## Abstract

*Objective*. To investigate the effects of topical and subconjunctival tigecycline on the prevention of corneal neovascularization.* Materials and Methods*. Following chemical burn, thirty-two rats were treated daily with topical instillation of 1 mg/mL tigecycline (group 1) or subconjunctival instillation of 1 mg/mL tigecycline (group 3) for 7 days. Control rats received topical (group 2) or subconjunctival (group 4) 0.9% saline. Digital photographs of the cornea were taken on the eighth day after treatment and analyzed to determine the percentage area of the cornea covered by neovascularization. Corneal sections were analyzed histopathologically.* Results*. The median percentages of corneal neovascularization in groups 1 and 3 were 48% (95% confidence interval (CI), 44.2–55.8%) and 33.5% (95% CI, 26.6–39.2%), respectively. The median percentages of corneal neovascularization of groups 1 and 3 were significantly lower than that of the control group (*P* = 0.03 and *P* < 0.001, resp.). Histologic examination of samples from groups 1 and 3 showed lower vascularity than that of control groups.* Conclusion*. Topical and subconjunctival administration of tigecycline seems to be showing promising therapeutic effects on the prevention of corneal neovascularization. Furthermore, subconjunctival administration of tigecycline is more potent than topical administration in the inhibition of corneal neovascularization.

## 1. Introduction

Corneal neovascularization can be a result of various conditions, including chemical injury, infectious, traumatic, toxic, and metabolic disease. The formation of new corneal blood vessels in corneal tissue leads to loss of corneal transparency which results in decreased visual acuity [1, 2].

Many mediators accelerate new vessel formation such as cytokines inflammatory mediators, various growth factors, tumor necrosis factor, vascular endothelial growth factor (VEGF), and matrix metalloproteases [3–6].

Various pharmacotherapies have been investigated for corneal neovascularization inhibition. Topical anti-VEGF and steroids remain the most commonly utilized treatments for suppressing angiogenesis in the cornea [2]. However, longer-term use of these drugs can lead to various adverse side effects, such as corneal thinning and delayed corneal epithelial healing [7].

MMP-9 is a proteolytic enzyme which degrades extracellular matrix connections and facilitates the migration of endothelial cells to other areas, leading to the formation of new vasculature. Therapies inhibiting MMP-9 are thus believed to have potential for inhibiting neovascularization. Previously, it has been shown that tetracycline and its derivatives inhibit corneal neovascularization via suppressing MMP-9 activity, a role that is distinct from its antimicrobial activity [8–11]. Tigecycline is a newer and more powerful agent of the tetracycline family, which is available as an injectable antibiotic [12]. It is expected that tigecycline inhibits corneal neovascularization but to date, the potency of tigecycline in the treatment corneal neovascularization has not been reported. Therefore, it is worthwhile to investigate the effect of tigecycline on cornea neovascularization so it can be combined with other antiangiogenic drugs.

The aim of this study is to investigate the effects of topical and subconjunctivally applied tigecycline on the prevention of experimentally induced corneal neovascularization in the rat model.

## 2. Materials and Methods

Thirty-two Wistar-Albino rats without corneal lesions and weighting 250 to 300 gram were used in the study. The rats were divided into 4 experimental groups with 8 rats in each group. All animals were housed in individual cages and maintained under standard conditions. The rats were placed in plastic cages in a temperature-controlled room in which a 12-12-hour light-dark cycle was maintained (07:00–19:00 hour light). This study was performed at Konya Training and Research Hospital, Department of Ophthalmology, Konya, Turkey. Animals were treated in accordance with the Association for Research in Vision and Ophthalmology's Statement for the Use of Animals in Ophthalmic and Vision Research.

### 2.1. Chemical Cauterization

The rats were anesthetized with a combination of ketamine hydrochloride (50 mg/kg) and xylazine (5 mg/kg). Eyes were topically anesthetized with 0.5% proparacaine hydrochloride. Induction of corneal neovascularization was performed by using a silver nitrate cauterization technique described by Mahoney and Waterbury [13]. All groups were chemically cauterized with an applicator stick coated with 75% silver nitrate and 25% potassium nitrate (Hemo Stop; Hizmet Medical, Istanbul, Turkey) to the central cornea for 10 seconds (with a diameter of 2 mm) under the operating microscope. Corneas and fornices were then washed with isotonic saline solution. To increase the reproducibility of the injuries, a single investigator cauterized all animals.

The severity of the chemical burn was evaluated 24 hours after cauterization. The extent of burn stimulus response was scored on a scale of 0 to +3 in accordance with the experimental model designed by Mahoney and Waterbury to test burn responses [13]. For each eye, the extent of burn stimulus response was scored as 0 (no blister, not raised above corneal surface), +1 (small blister, raised slightly above the surface), +2 (medium blister, raised moderately above the surface), and +3 (large blister). Only the corneas with a burn stimulus score of +2 or higher were included for the calculation of the mean burn stimulus and neovascularization scores in each group. Treatment started immediately after cauterization in the 4 groups and only the right eyes were treated immediately with applications. All procedures were performed by the same investigator.

### 2.2. Grouping

Thirty-two rats were randomly assigned to 1 of 4 groups with 8 rats in each group. Group 1 received 1 mg/mL of tigecycline (Tygacil, PFIZER) topically. In one study, 1 mg/mL topical application of doxycycline which is tetracycline derivatives was found effective for inhibiting corneal neovascularization [14]. As the systemic dose rates of tigecycline and doxycycline are the same in humans, the dose of topical tigecycline was selected to be the same as the topical drugs application of doxycycline. Group 2 received topical 0.9% saline as a control group. The drops were applied topically twice a day for 7 days. Group 3 rats were treated with a 0.05 mL subconjunctival injection of 1 mg/mL tigecycline. The subconjunctival dose of tigecycline was the same as the topical dose. Group 4 rats were treated on a daily basis with a subconjunctival injection of 0.05 mL 0.9% saline as a control group. The rats were anesthetized prior to the subconjunctival injections. Subconjunctival injections were performed using a 30-gauge needle by using an operating microscope and inserted 2 mm posterior to the limbus at the superior bulbar conjunctiva.  Table 1 summarizes the information of the groups.

### 2.3. Measurement of Corneal Neovascularization

In each experimental group, photographs were taken on the eighth day after treatment. Anterior segment photos were taken with a Topcon digital camera (BG-4 model) mounted on biomicroscopy (Topcon, DC-3, Tokyo, Japan) to determine the extent of corneal neovascularization. The percentage of the portion of neovascularized cornea was calculated using the software program Image J 1.46 (by Wayne Rasband at the Research Services Branch, National Institute of Mental Health, Bethesda, MD, USA). The number of pixels showing neovascularization was expressed as a percentage of the entire corneal pixel number [15, 16].

After the photographs were taken, all the animals were sacrificed. The eyes were enucleated and the globes were fixed in fresh prepared 4% paraformaldehyde. After fixation for 24 hours, corneal samples were prepared by macroscopic incisions from limbus to limbus passing through central cornea in order to include the region with the highest neovascularization intensity. Thereafter, fixed tissues were sectioned serially in the horizontal plane at a thickness of 4 *µ*m. In most sections, the density of neovascularization was obtained from the central region of the cornea. The sections were stained with hematoxylin and eosin. The degree of corneal neovascularization was evaluated histomorphometrically with light microscopy. Images were digitally captured and the density of neovascularization was analyzed quantitatively in areas of maximum density.

### 2.4. Scoring of Hematoxylin-Eosin Staining

To evaluate the neovascularization intensity histomorphometrically, digitally saved micrographic images from the light microscope were used. A point grid was put on the screen where the density of neovascularization was maximum in the corneal tissue. The numbers of neovascularization refer to the total density for that sagittal section in an area where neovascularization were found. The grid size on the image was scaled to real size for estimation of maximum density of neovascularization (No/mm^2^) [17, 18]. The calculations were made by two independent histologists in a double-blinded design.

Data were given as the median and 95% confidence interval. Statistical analyses were performed using SPSS software version 15.0 for Windows (SPSS, Chicago). The Mann-Whitney-*U* test was used for comparisons between the administration methods and control groups. The Kruskal-Wallis analysis of variance test was performed for multiple comparisons of groups. If the Kruskal-Wallis analysis of variance test was significant, then pair-wise comparison of groups was carried out to determine the difference. To account for multiple comparisons, adjusted *P* values were taken into consideration. Differences were considered statistically significant when *P* values were less than 0.05.

## 3. Results

**Figure 1:**
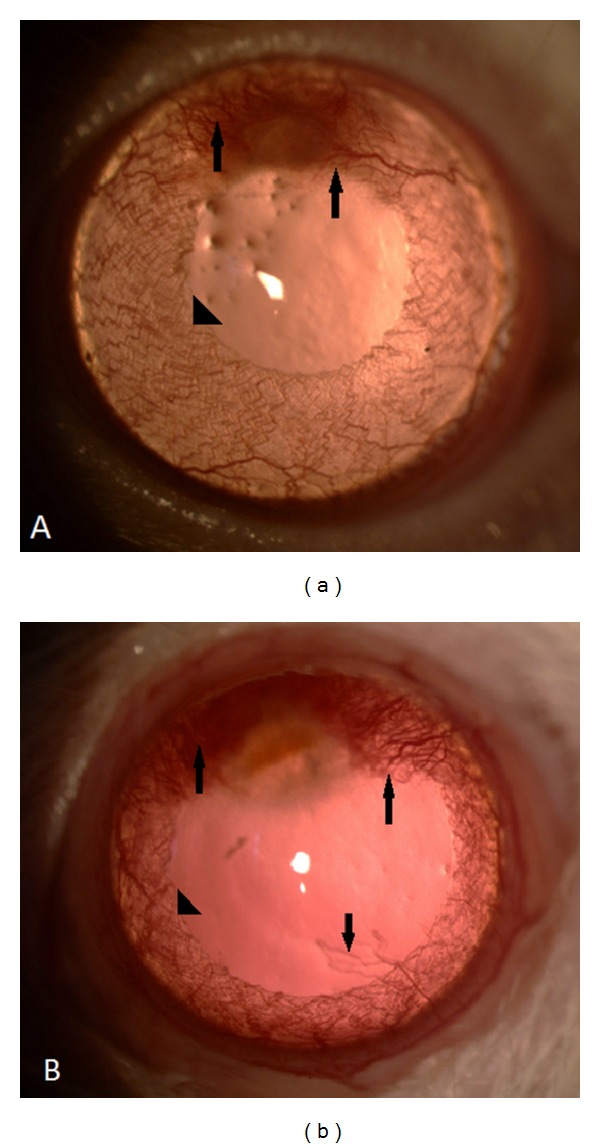
Biomicroscopic corneal findings of the cornea 7 days after induction of corneal burn in eyes. Arrows describe corneal neovascularizations; arrowheads describe the vessels of albino-rat iris. (a) An example of tigecycline-treated eyes. Presence of fewer vessels on the cornea than in control group. (b) An example of control eyes.

There were no statistically significant differences in the burn stimulus scores of each cornea in the study groups and control groups (*P* > 0.05). All 32 eyes had a reasonable degree of corneal neovascularization on day 7 after the chemical burn. No animal developed corneal perforation. The corneal photographs with neovascularization of the groups after the chemical burn were shown in Figure 1.

**Figure 2:**
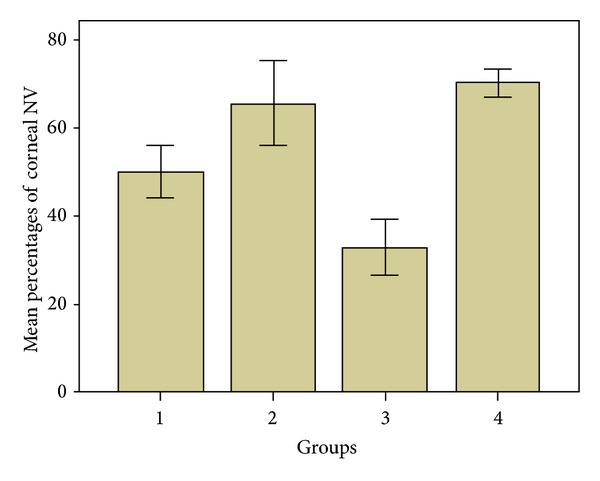
The percentage of corneal neovascularization by groups. Subconjunctivally tigecycline-treated eyes (group 3) showed significantly less corneal neovascularization than other groups.

The median percentages of corneal neovascularization are presented in Figure 2 and Table 2. The median percentages of corneal neovascularization in groups 1 and 3 (the study groups) were 48% (95% confidence interval (CI), 44.2–55.8%) and 33.5% (95% CI, 26.6–39.2%), respectively. The median percentages of corneal neovascularization in groups 2 and 4 (the control groups) were 67% (95% CI, 55.8–75.2%) and 70% (95% CI, 67.3–73.4%), respectively. The median percentages of corneal neovascularization of groups 1 and 3 were significantly lower than that of the control group (*P* = 0.03, *P* < 0.001, respectively). When groups 1 and 3 were compared with each other, group 3 showed significantly lower corneal neovascularization when compared with group 1 (*P* = 0.001).

**Figure 3:**
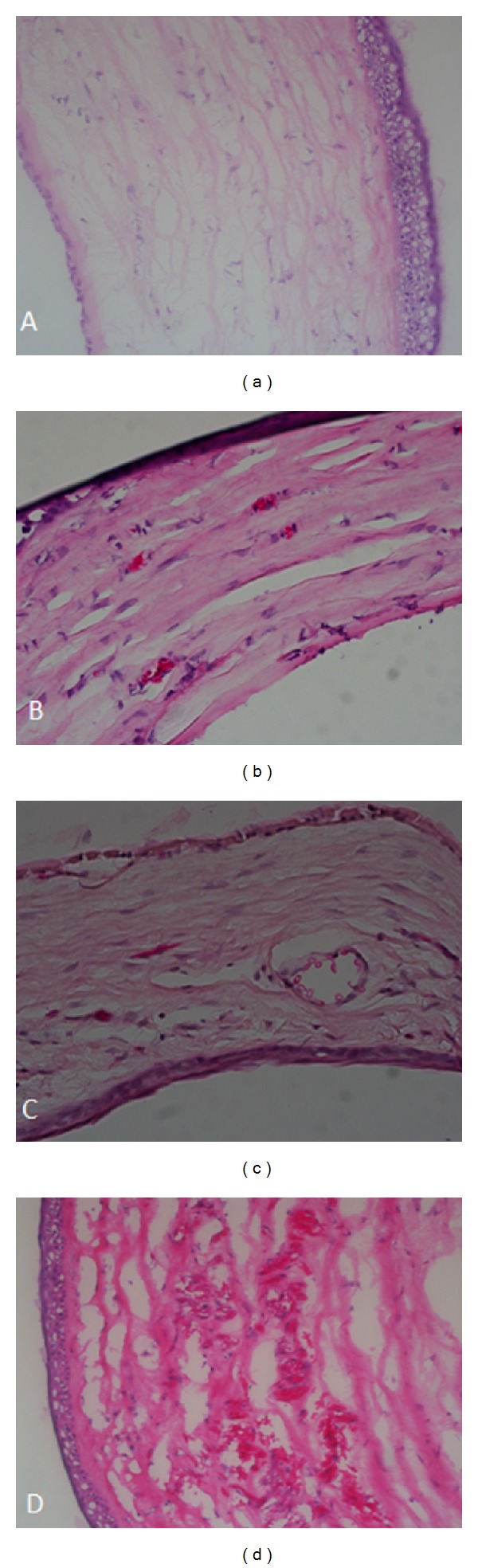
Histopathologic photographs of cornea after chemical burn. (a) Normal cornea. (b) An example of topically tigecycline-treated eyes revealing less corneal neovascularization. (c) Subconjunctivally tigecycline-treated eyes showing virtually few neovascularization than in control group in stroma. (d) Diffuse and intense neovascularization affecting deep stromal layers after in control eyes.

Figure 3 illustrates histopathological findings. Maximum density of neovascularization in each group as determined by histopathology is presented in Table 2. Neovascularization intensity in study groups was significantly lower than the control groups with respect to the density of neovascularization. No local or systemic adverse effects were seen from either treatment group.

## 4. Discussion

We examined here for the first time the therapeutic efficacy of tigecycline for the inhibition of corneal neovascularization. In this study, corneal neovascularization was reduced significantly following topical and subconjunctival administration of tigecycline. The effectiveness of the subconjunctival route is greater than for the topical administration of tigecycline. Perhaps a sufficient dose was not administered topically. A higher dose or more frequent injections of tigecycline may need to be given topically and future studies may focus on tigecycline dose. Additionally, we used subconjunctival injection on a daily basis which may cause tigecycline levels in that region to be maintained for a sufficient time and at a sufficient concentration.

Several reports have demonstrated the clinical efficacy of derivatives of the tetracycline family on the reduction of corneal neovascularization via downregulation of MMP-9 expression [19–23]. However, in the literature no clinical studies have investigated the clinical efficacy of tigecycline for inhibiting corneal neovascularization. The mechanism of action of tigecycline on the corneal neovascularization treatment may be due to its inhibitory effects on the activity of MMP-9. VEGF and MMP-9 are potent regulators of angiogenesis which play a key role in corneal tissue with angiogenesis [24]. Several studies showed that the MMP release biologically active VEGF from the extracellular compartments [25–28]. This effect of tigecycline on VEGF may enhance the therapeutic effects of anti-VEGF which may play an important role in inhibiting corneal neovascularization. So it is expected that the combination of various angiogenesis inhibitors and tigecycline might have better therapeutic benefits. Su et al. showed that doxycycline treatment reduces MMP and VEGF expression [11]. VEGF might have a role in maintaining normal corneal function and/or epithelial healing [29]. Current anti-VEGF therapies, although efficacious, require prolonged treatment regimens which may cause various ocular complications such as a prolonged corneal epithelial healing period and increased the occurrence of corneal ulceration. Recently, several studies have shown that topical application of bevacizumab led to corneal thinning and delayed corneal wound healing [7, 30]. Therefore, it may be worthwhile combining anti-VEGF and tigecycline so angiogenesis can be inhibited via different pathways. This may have a more desirable therapeutic benefit and reduce anti-VEGF side effects for clinical application.

The limitations of our study are that our study does not show the ideal concentration of tigecycline to inhibit rats corneal neovascularization. Therefore, different doses must be studied. Further animal studies are needed to further optimize the tigecycline dose for treating corneal neovascularization and to characterize any side effects associated with treatment. VEGF and MMP-9 expression may also need to be examined with western blotting and polymerase chain reaction analysis to assess the antiangiogenic mechanisms of tigecycline.

In summary, tigecycline seems to be promising therapeutic effects on the prevention of corneal neovascularization. Furthermore, subconjunctival administration of tigecycline is more potent than topical administration in the inhibition of corneal neovascularization. Tigecycline can be combined with other antiangiogenic drugs as an alternative treatment for the clinical management of corneal neovascularization. However further investigation is needed to the usefulness of tigecycline for treating corneal neovascularization in the clinical setting.

## Figures and Tables

**Table 1:** Summary of groups information.

Groups	Drug	Concentration	Administration	Volume	*n*
Group 1	Tigecycline	1 mg/mL	Topical	50 *µ*L	8
Group 2	Saline	0.9%	Topical	50 *µ*L	8
Group 3	Tigecycline	1 mg/mL	Subconjunctival	0.05 mL	8
Group 4	Saline	0.9%	Subconjunctival	0.05 mL	8

**Table 2:** The median percentages of corneal neovascularization and maximum density of neovascularization (histopathological examination) in groups.

	Group 1 Topical tigecycline	Group 2 Topical 0.9% saline	Group 3 Subconjunctival tigecycline	Group 4 Subconjunctival 0.9% saline
Neovasculized area (CI)	48 (44.2–55.8)	67 (55.8–75.2)	33.5 (26.6–39.2)	70 (67.3–73.4)
Maximum density of neovascularization (CI)	35.5 (21.6–49.4)	66.5 (47.5–8.2)	19.5 (12.3–30.2)	57 (36.4–85.6)

Results were defined as median values (95% confidence intervals (CI)).
